# A Look to Future Directions in Gene Therapy Research for Monogenic Diseases

**DOI:** 10.1371/journal.pgen.0020133

**Published:** 2006-09-29

**Authors:** Matthew H Porteus, Jon P Connelly, Shondra M Pruett

**Affiliations:** University College London, United Kingdom

## Abstract

The concept of gene therapy has long appealed to biomedical researchers and clinicians because it promised to treat certain diseases at their origins. In the last several years, there have been several trials in which patients have benefited from gene therapy protocols. This progress, however, has revealed important problems, including the problem of insertional oncogenesis. In this review, which focuses on monogenic diseases, we discuss the problem of insertional oncogenesis and identify areas for future research, such as developing more quantitative assays for risk and efficacy, and ways of minimizing the genotoxic effects of gene therapy protocols, which will be important if gene therapy is to fulfill its conceptual promise.

## Introduction

With the recognition that thousands of diseases are caused by changes in single genes, so-called monogenic diseases, and the advent of recombinant DNA technology, the idea that one could manipulate the nucleic acid content of diseased cells to cure disease, “gene therapy,” was born. In 1978, David Baltimore summarized the promise of gene therapy and the optimism that many felt:

. . . it would be a triumph of medicine if the effects of such genes could be countered. . . . One approach involves altering some cells of the body so that they can carry out the needed function. A patient could, for instance, be treated in this way for a blood disease caused by an abnormal protein made by a mutant gene. A normal gene would be inserted into the precursor cells—immature bone marrow cells that ultimately develop into functioning blood cells. In this way, a normal protein could be made in place of, or along with, the aberrant protein. The genetically altered blood cell precursor could then cure the patient's disease. . . . It is likely to be the first type of genetic engineering tried on human beings, and might be tried **within the next five years**. [1]

In the eyes of the public, general scientists, and practicing physicians this potential remains unrealized. While progress has not occurred as fast as anticipated, the strategy that Dr. Baltimore outlines remains one of the working paradigms in the field and would still be a “triumph of medicine.” This review briefly examines recent progress in the field and looks forward to areas of study that we perceive as being essential so that 28 years from now gene therapy is not seen as something that once had great promise. We will discuss the importance of developing more quantitative assays for both efficacy and risk, and ways to minimize the risk of gene therapy protocols. A major focus of gene therapy research has been, and will continue to be, the development of viral vectors for gene delivery, including novel adenoviruses, adeno-associated viruses, herpes simplex viruses, and retrovirus (including foamy and lentiviruses). A thorough discussion of these vector delivery systems is beyond the scope of this review, and comprehensive discussions of the different viral vectors used in gene therapy can be found elsewhere [2–4].

## Summary of Recent Clinical Trials of Gene Therapy for Monogenic Diseases

In the last decade there have been four monogenic diseases for which seminal gene therapy trials have been conducted: ornithine transcarbamylase (OTC) deficiency, hemophilia caused by factor IX deficiency, severe combined immunodeficiency (SCID), and chronic granulomatous disease (CGD), the results of which are briefly summarized in Table 1 [5–12]. Significant attention has been paid to the serious adverse events that have come from these trials, and in fact we use these adverse events as a basis for formulating future areas of research in gene therapy. It must be highlighted, however, that an increasing number of patients, particularly those with SCID, have benefited from participating in these gene therapy trials. Moreover, for most of these diseases, contemporary therapy is either inadequate or associated with serious adverse events themselves. Evaluation of the gene therapy trials must be done in the context of currently available treatments. Finally, as with any new therapy, for example the development of bone marrow transplantation and solid organ transplantation, there will be growing pains. It would be naively unrealistic to expect that gene therapy will not suffer similar pains. These growing pains have taught us about new obstacles that need to be solved and include the problems with in vivo administration of viral vectors and the problem of insertional oncogenesis.

**Table 1:**
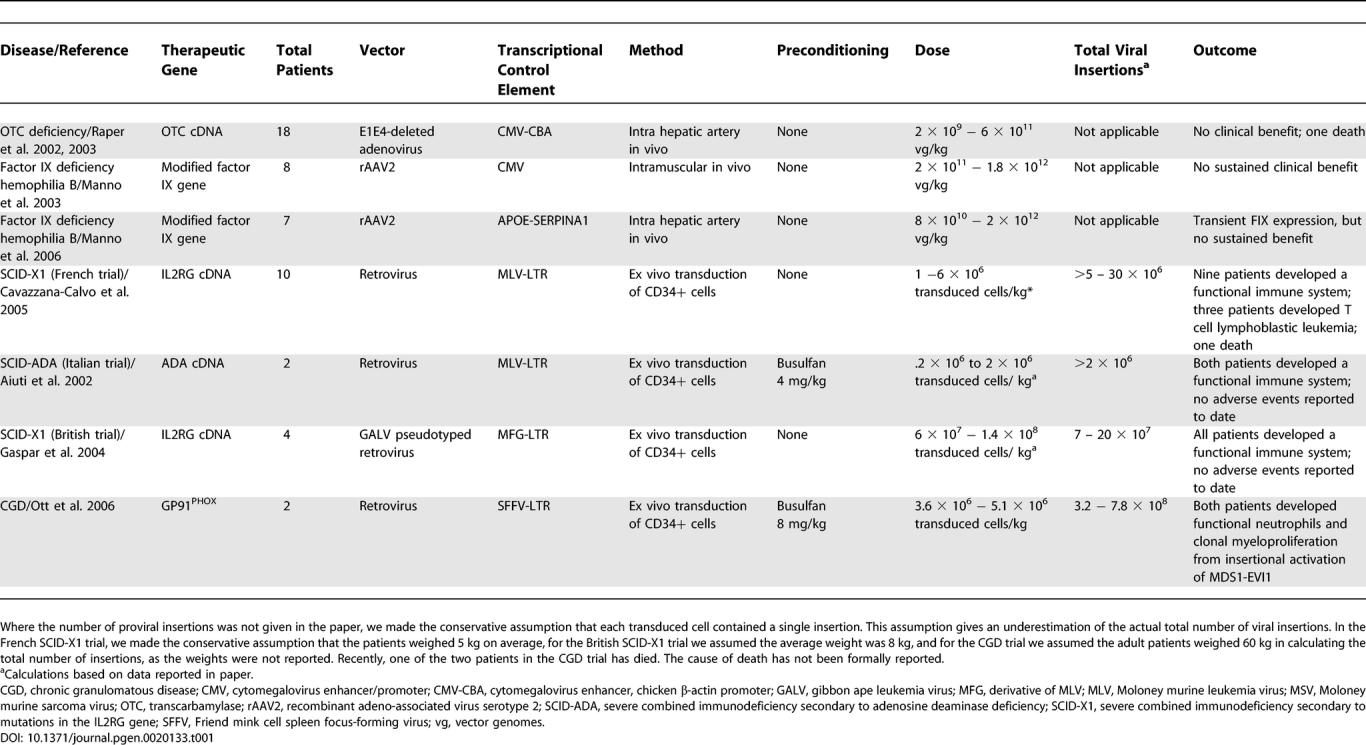
Summary of Recent Human Gene Therapy Trials

## Problems with In Vivo Administration of Viral Vectors

A **first** problem is the immune response to in vivo–administered viral vectors. Many viral proteins are capable of eliciting an immune response, the magnitude of which may be difficult to predict and varies from patient to patient. In the OTC trial, this variable inflammatory response resulted in the death of one patient; and in the hemophilia trials, the immune response eliminated virally transduced cells thereby abrogating any potential benefit. Transient or long-term immunosuppression is a possible solution to this problem which we will not discuss further here.

A **second** problem with in vivo administration of viral vectors is that multiple cell types are exposed to the risks of being infected with the administered virus. In the in vivo administration of viral vectors, certain cell types are the desired target because they have the greatest chance of giving clinical benefit. But multiple other cell types, which have a low likelihood of contributing to clinical effectiveness, are exposed to potential infection with the viral vector and the possible deleterious consequences, such as activation of the inflammatory response or insertional oncogenesis if an integrating viral vector is used.

A **third** problem with the in vivo administration of viral vectors is the risk of germ line transduction. While germ line transduction has not been observed so far, if inadvertent germ line transduction did occur, it could create a public outcry against gene therapy in general.

## Insertional Activation of Proto-Oncogenes

The SCID and CGD trials have been the first “successful” gene therapy trials, as some patients have benefited from the procedure. Nonetheless, the French SCID trial and the CGD trial have also shown that the insertional activation of proto-oncogenes is a serious problem that can result in either leukemia or a clonal myeloproliferative condition [9,12]. In both of these trials, a murine leukemia derived replication-incompetent retroviral vector was used to infect purified CD34^+^ cells with the transgene being driven by the retroviral long terminal repeat (LTR). In the French SCID trial, three patients developed leukemia between two and three years after the infusion of transduced cells from the activation of the LMO-2 proto-oncogene [9,13]. In the CGD trial, the insertional activation of the MDS1-EVI1 genes (EVI1 is a transcription factor involved in the development of some myeloid leukemias [14]) may have contributed to clinical benefit but also led to a clonal myeloproliferation resembling a pre-leukemic state [12]. Whether these patients will develop leukemia or myelodysplasia remains to be seen. For both trials, preclinical studies were neither robust nor quantitative enough to detect the risk of the insertional oncogenesis. Subsequent murine studies have revealed that the risk of developing T cell leukemia from retroviral activation of the LMO-2 proto-oncogene might have been anticipated [15–17].

One of the interesting observations from these human trials is that the clonal disorders arose in the cell lineage for which there is an underlying defect (T cells in the SCID trial, myeloid cells in the CGD trial) and not in lineages that did not have a disorder (for example, there has been no evidence of myeloproliferation in the SCID trials). The underlying reason for this phenomenon is not clear but may relate to an interaction between the transgene expressed, the integration site of the viral vector, and the proliferation of the particular cell type. Understanding these relationships has important ramifications for the future of gene therapy research and for our understanding of the oncogenic process in general.

## Oncologic Principles Relevant to Gene Therapy

The nature of the oncogenic process has been an area of intense study longer than the existence of the field of gene therapy. It is now apparent that these two fields, in addition to the evolving field of stem cell biology, are intertwined. Through the studies of numerous investigators, including Bishop and Varmus who proved that cancer is a genetic disease, Knudson who proposed the “two-hit” model of oncogenesis, Ashley who mathematically deduced the multi-hit nature of oncogenesis, Vogelstein and Kinzler who proposed the multi-step model for cancer, and Weinberg and Hahn who defined critical molecular aspects of these hits, cancer is now understood to require on the order of four to six genetic lesions [18–22]. Studies of somatic genomic mutation rates show that the acquisition of genetic lesions is a continuous and cumulative process and that one in three people will develop enough hits in a single cell by the age of 80 to develop cancer. People who inherit a single “hit” in a tumor suppressor gene such as p53 (Li-Fraumeni syndrome) have a lifetime risk of developing cancer of 100%. Cancers in Li-Fraumeni patients can take months to years to decades to develop. Furthermore, studies of pediatric acute lymphoblastic leukemia show that the leukemic clone can be present at birth and not manifest itself as a life-threatening disease until years later [23,24]. Thus, observing a mouse for several months is not sufficient to assess the oncogenic risk for a particular protocol. Any process that speeds the creation of genetic hits, such as viral insertions, will hasten the development of cancer.

Gene therapy strategies employing the use of many viral vectors, such as retroviral vectors, are based on the uncontrolled integration of a therapeutic gene. A fundamental problem with this strategy is that hundreds of millions of cells are infused, each of which has acquired at least one genetic hit. Even those viruses that are “non-integrating,” such as recombinant adeno-associated virus (rAAV), have a low rate of random integration that in clinical gene therapy trials could potentially cause billions of insertional mutations [25]. These hits are likely to be more problematic than random mutations, as viral vectors preferentially integrate into the coding or regulatory regions of expressed genes, often resulting in upregulation of the genes into which they integrate [25–27]. Thus, it is almost certain that multiple proto-oncogenes will be activated in any retroviral or lentiviral gene therapy protocol. It is also likely that insertional mutagenesis may inactivate one allele of a number of tumor suppressors. Although this inactivation is not likely to result in cancer in the short-term, it may lead to an increased risk of cancer years to decades later—when those cells will have had the time to acquire additional mutations through the normal ravages of life. Finally, an increased risk of cancer is associated with increased proliferation. Since almost all gene therapy protocols envision expansion and proliferation of transduced cells as an important component to clinical efficacy, this also increases the risk of subsequent oncogenesis.

This somewhat pessimistic assessment highlights that significant challenges remain for gene therapy. Conquering these challenges will require more quantitative ways to assess both risk and efficacy and novel approaches to improve the risk–benefit ratio. We expect that as any new advance is made that might improve the risk–benefit ratio, including those discussed below, the improvement must be assessed quantitatively.

## Developing Quantitative Models of Cancer Risk Assessment for Gene Therapy Protocols

The preclinical assessments of the safety of gene therapy protocols have been inadequate in giving a preclinical assessment of the risk of oncogenesis in human studies. These preclinical safety models have been inadequate for three reasons: 1) analysis of an insufficient number of integration events, 2) observation for an insufficient length of time, and 3) subjective rather than quantitative assessment of risk.

Most preclinical studies of gene therapy are performed in adult mice, the average weight of which is 20 g. Thus, giving a mouse 100,000 transduced cells (5 × 10^6^ cells/kg) with an average of one integration event per cell (as was done by May et al. [28]) is exposing the mouse to the potential consequences of 100,000 insertional events. If a 70-kg person were to receive 5 × 10^6^ transduced cells/kg, they would be exposed to the consequences of 350,000,000 insertional events. If we assume that each insertional event has an equal probability of causing cancer, then it would require studying 3,500 mice to assess the cancer risk for a single patient. If we wanted to assess the cancer risk per 100 patients, we would have to assess the cancer risk of 350,000 mice.

Even if the appropriate number of insertion events had been studied in mice, wild-type mice remain an inadequate model for the assessment of oncogenic potential because of their short lifespan. Mice live, on average, two years. As discussed above, the leukemias that developed in the French SCID trial took more than two years to develop, and the studies of the ontogeny of pediatric acute lymphoblastic leukemia also show that it can take much longer than two years for a leukemia to become apparent [9,23,24]. Thus, the standard mouse model is inadequate for the quantitative assessment of oncogenic risk.

Since all medical interventions carry some risk, it is incumbent on gene therapy researchers to establish quantitative measures of this risk. For example, it may be found for a certain disease that one million cells with an average of 1.3 integration events per cell per kilogram are needed for efficacy (or a total number 100 million (10^8^) insertion events). If the risk of oncogenic transformation per integration event is 10^−10^, then the risk of creating cancer is 1 in 100 and the risk–benefit ratio supports using gene therapy. If, on the other hand, the risk of oncogenic transformation per integration event is 10^−9^, then the risk of creating cancer is 1 in 10 and the risk–benefit ratio may not favor using gene therapy. Of course, such decisions depend on the prognosis for the disease, the risk–benefit ratios for alternative treatments, and we use these reasonable numbers for illustrative purposes.

## Proposed Quantitative Models for Assessment of Risk

There are many possible methods to quantitate the risk of a gene therapy protocol. These assays will evolve as our understanding of oncogenesis improves and other possible adverse events of gene therapy are revealed. We believe these quantitative risk assessments should be performed not just with current viral-based strategies but also with ones in the future that may include the use of potentially improved viral vectors, insulators, the use of targeted integration such as by phiC31 integrase or zinc finger nucleases (ZFNs), and manipulated stem cells.

We envision two general types of assays to assess the oncogenic risk of a gene therapy protocol: cell-based and mouse-based. Cell-based assays, such as the 3T3 transformation assay, are well-established and standardized. 3T3 cells are murine fibroblasts that have escaped senescence and passed through crisis but still retain characteristics of nontransformed cells, such as contact inhibition and the inability to grow as colonies in soft agar. A standard procedure to assess the oncogenic property of a gene, a gene variant, or a chemical compound is to determine the rate that it transforms 3T3 cells into cells that can grow in soft agar. A gene therapy protocol could be tested for its ability to transform 3T3 cells. Ideally, the protocol would not transform 3T3 cells above the background rate. Once that is achieved, the gene therapy protocol could be more rigorously evaluated by determining the rate it transforms 3T3 cells that have been further sensitized to transformation, such as by co-treatment with mutagenic chemicals or co-transformation with a weak oncogene. By using sensitized 3T3 cells, subtle differences between gene therapy protocols may be revealed.

The second way gene therapy protocols should be tested is using quantitative animal models. Ideally, one would like an animal model that in one to two years could accurately predict the oncogenic risk in a human for 50 years. As discussed above, standard mouse models are inadequate for this purpose, and such an ideal model does not currently exist. An alternative is to use mouse models that have been sensitized to tumor development (cancer susceptibility model). For example, if one were interested in studying the oncogenic risk of a hematopoietic-based gene therapy protocol, murine hematopoietic cells deficient in p53 could be tested. When p53^−/−^ hematopoietic cells are transferred to isogenic p53 wild-type mice, the mice develop lymphoma with a latency of about 2 mo [29]. Ideally, one would find that even if 100 million integration events (a hypothetical number of events determined to be needed for clinical efficacy for that disease) were transferred into p53 deficient cells, the latency to lymphoma development would not change. The protocol could be more rigorously tested by examining the latency to lymphoma after further sensitizing the cells by treatment with a chemical mutagen or co-transforming with a weak oncogene. If in these models tumors were induced at a rate 100-fold lower than that needed for clinical efficacy it would provide strong preclinical evidence that the protocol was safe for human trials. Such a model is being used to compare the rates of transformation by MLV- and HIV-based vectors using Cdk2a^−/−^ hematopoietic stem cells [13]. We have outlined one such strategy to test the safety of a gene therapy protocol in animals, but there are many different models one could use, and these different models may be more appropriate for different gene therapy protocols. Establishing a uniform animal model for testing would be beneficial because it would allow different gene therapy protocols to be directly compared. Moreover, it would be useful to test the gene therapy protocols that have already been used in human trials to correlate the rate of transformation in an animal model to what was found in the human trials, thereby establishing a baseline for future studies.

In summary, quantitative assays for safety should be developed. We have proposed potential cell-based and mouse-based assays, but it is likely that researchers can develop better quantitative cell- and animal-based models that more accurately assess the oncogenic risk specific to each gene therapy protocol. We acknowledge that the studies outlined above set a high bar for gene therapy research. By setting such a bar, however, we will retain the trust of the patients, the public, and the funding agencies on which our work depends.

## Quantitative Measures of Efficacy

If the measure of risk is defined on a per event basis, then to rationally balance risk–benefit, quantitative measures of efficacy per event must also be developed. To illustrate this principle, we will use hemophilia B (factor IX deficiency) as an example. To convert severe hemophilia to mild hemophilia requires generating a circulating level of factor IX of 5% (or 250 ngs/ml or approximately 500,000 nanograms per total circulating volume for an average adult). If a particular vector could, on average, generate 10 picograms per event, then to achieve clinical efficacy one would expect 50 million events to be necessary. If, on the other hand, another vector gave 10 times as much factor per average event, then 10-fold fewer events would be necessary and this improvement in efficacy might also translate into improved safety. May et al. (2000), for example, used such a comparison to demonstrate that a β-globin–expressing lentivirus with longer hypersensitivity regions from the β-globin locus control region was better than a vector with shorter hypersensitivity regions [28]. It is likely that for each disease, different quantitative measures will have to be established, but once these measures are established, they will allow rational comparison of different vector systems and protocols.

## Approaches to Minimizing the Oncogenic Risk of Gene Therapy

To improve the risk–benefit ratio of gene therapy, it is important to continue to develop ways to minimize the risk of insertional oncogenesis. Four possible ways to minimize the risk are: 1) continued improvements in vector design, 2) buffer the genome from the effect of viral integrations, 3) develop ways to control transgene integration, or 4) develop ways to expand a small number of genetically characterized modified stem cells in vitro and then re-infuse these non-oncogenic cells into the patient.

## Improving Safety through Modified Vectors

In the two gene therapy trials that caused proliferative diseases, the investigators used a retroviral vector using the viral LTR to drive expression of the transgene. In the last several years, different vectors have been formulated, such as self-inactivating vectors and lentiviral vectors, which may have improved safety. In addition, improvements in safety may be gained by using tissue-specific promoters to drive transgene expression. These tissue-specific promoters may give increased transgene expression thereby increasing the efficacy per given event, and thus fewer events would be needed for clinical efficacy. By using more specific promoters, more tightly regulated expression may be obtained that might also minimize the potential oncogenic properties that overexpression may entail. In retroviral and lentiviral vectors, however, the LTR would still be present and could still potentially act as an enhancer for neighboring genes. It is also possible that more specific promoters will not confer significant safety advantages. In the SCID and CGD trials, for example, insertional activation of a proto-oncogene occurred in a cell-type–specific manner and not in a heterologous cell type.

An alternative vector system is to use transposons as a nonviral delivery method. Transposon-based delivery systems have been shown to work in a wide variety of cell types, including stem cells [30]. In their current formulations, however, the integration events remain uncontrolled and thus may suffer the same problems of integration-based viral delivery systems. In fact, transposon-based insertions are being developed as a tool for cancer gene discovery [31]. An active but still preliminary area of research is to try to create hybrid transposases to target transposon integration to defined regions of the genome.

## Buffering the Genome Using Insulators

Viral integrants under the control of LTR promoters can activate genes near the insertion site [9,12,32]. Buffering the genome surrounding the insertion site might be beneficial in minimizing such effects. Insulators are small DNA elements that act as barriers thereby preventing promoter–enhancer elements and/or chromatin modifications from influencing the expression of neighboring genes [33]. The cHS4 insulator, derived from the chicken β-globin locus, is the insulator being studied most intensely for gene therapy purposes. When insulators are incorporated into integrating retroviral vectors being developed for gene therapy purposes, position-effect variegation is decreased and the amount of transgene expressed is increased for each insertion [34–36]. It is hoped that insulators will protect the surrounding genome from insertional activation from retroviral LTRs. From the structural organization of such vectors, however, one would only expect a decrease but not an elimination of LTRs acting as enhancers on neighboring genes. Studies directly testing the degree to which insulators protect the surrounding genome from LTRs are ongoing, the publication of which is much anticipated. While insulators may protect the genome from the insertional activation of proto-oncogenes, they may be limited because they will not protect against the insertional inactivation of tumor suppressors by viral integrations.

## Minimizing the Risk by Targeting Transgene Insertion

An alternative to buffering the genome from an uncontrolled integration site is to target integration to “safe” genomic locations. Currently there are two possible methods being developed to target transgene integrations: the use of the phage C31 integrase and homologous recombination.

### 

#### Targeting integration with φC31 integrase.

Michelle Calos and colleagues have developed vectors based on the φC31 integrase [37]. The *Streptomyces* phage integrase C31 catalyzes efficient site-specific recombination between two relatively short (~30–40 bp) sequences. In the natural phage life cycle, the temperate phage infects bacteria and integrates into the host genome. To accomplish integration, the phage encodes φC31 integrase, which catalyzes recombination between the phage DNA through the phage attachment site, *attP,* and the bacterial DNA through the bacterial attachment site, *attB.* In mammalian cells, φC31 has been used to mediate integration of plasmids encoding the *attB* site into a limited number of native sequences bearing partial sequence homology with the *attP* sites called pseudo *attP* sites.

Recent studies using vectors based on the φC31 integrase have been able to demonstrate stable genomic integration, prolonged expression of therapeutic genes, and have shown efficacy in models of tyrosinemia type I [38], epidermolysis bullosa [39], factor IX deficiency [40], and SCID-X1 [41]. While targeting transgene integration with φC31 is showing promise, there are several remaining issues. One issue is whether the expression of the integrase causes genomic instability. Studies examining the integration specificity of the phage φC31 integrase into the human genome have estimated that there are between 100 and 1,000 pseudo-*attP* sites. There is concern and some evidence that the φC31 integrase may catalyze recombination between these pseudo-*attP* sites, thereby causing genomic instability [42]. To minimize these genotoxic effects, directed evolution is being used to modify the φC31 integrase to improve the specificity of its action to a more limited number of sites [43].

#### Using homologous recombination to minimize genotoxic effects.

A conceptually appealing way to treat monogenic diseases caused by small mutations is to use homologous recombination to correct the mutations while leaving the rest of the genome untouched. In diseases caused by larger mutations, such as the chromosomal inversion that causes almost 50% of the cases of severe Factor VIII deficiency, one could use homologous recombination to target the transgene to a defined genomic location. In mammalian cells, however, the spontaneous rate of homologous recombination is on the order of 10^−6^ and thus too low to be considered for gene therapy purposes [44,45]. Recently, two strategies have been found that increase the rate of homologous recombination to levels that might be of therapeutic use: the use of rAAV and the use of DNA double-strand breaks (DSB).

In 1998, Russell and Hirata reported that cells infected with rAAV underwent a high rate of homologous recombination [46]. Russell's group subsequently demonstrated that rAAV could correct a variety of lesions in a wide range of cell types [47]. The mechanism by which rAAV mediates high rates of gene targeting remains unclear but does seem to involve the cell's endogenous homologous recombination machinery. Building on this work, the Russell group then used rAAV-mediated homologous recombination in mesenchymal stem cells to inactivate a dominant mutation in the collagen Col1A1 gene that causes osteogenesis imperfecta [48]. By inactivating the dominant mutation, they demonstrated that the cells had phenotypic correction of the underlying defect. Several issues remain with rAAV-mediated homologous recombination. To obtain recombination rates of useful magnitude, infection of cells with high multiplicity of infections is required, increasing the incidence of random integration. In fact, most integration events will be nonhomologous rather than homologous. A study of these nonhomologous rAAV integration events showed that many were associated with chromosomal rearrangements and occurred in the control regions of genes [25,49].

A second method of stimulating homologous recombination is by inducing DNA DSBs in the target locus. In the mid-1990s a number of labs demonstrated that a specific DSB in a genomic target created by the I-SceI homing endonuclease stimulated homologous recombination between the genomic target and transfected plasmid (“gene targeting”) by 1,000-fold [50]. With optimization, gene targeting rates of 3%–5% can be obtained [44]. The limitation of this approach is that endogenous genes do not contain recognition sites for I-SceI or other homing endonucleases. If DSBs are to be used to stimulate gene targeting, a method to create site-specific DSBs needed to be developed. ZFNs have shown promise in being such a reagent. These nucleases are artificial proteins in which a zinc finger DNA binding domain is fused to the nuclease domain derived from the type IIS restriction enzyme FokI and were first developed by Chandrasegaran and his colleagues [51,52]. The first demonstration that ZFNs might be useful for gene therapy was in 2003 when Porteus and Baltimore demonstrated that model ZFNs could stimulate gene targeting by several-thousand–fold [44]. Urnov et al. (2005) then showed that ZFNs could be designed to recognize an endogenous target gene, in this case the IL2RG gene, and to create targeting rates of 20% in the human K562 cell line and 5% in primary human T cells [53]. These results provide optimism about the possibility of using ZFNs to stimulate homologous recombination for therapeutic purposes.

One of the appeals of ZFNs is that they can be modified to recognize novel target sequences. Theoretically it may be possible to make ZFNs to essentially any target sequence, but a current area of active research is to establish the best practical method to do this. Barbas and his colleagues have released a program that allows users to examine a sequence of interest for binding sites to which zinc fingers can be assembled (http://www.zincfingertools.org). Whether this relatively simple modular-assembly approach is good enough to make highly active and specific ZFNs or whether more complex selection-based approaches are needed remains to be determined.

In the continued development of ZFNs, it is important to demonstrate that ZFNs do not create oncogenic mutations. DSBs are known to be mutagenic and can lead to chromosomal translocations. Studies of ZFNs in model organisms and mammalian cells have demonstrated that they cause cytotoxicity by the creation of DSBs [44,54]. It is critical, therefore, to design ZFN systems that minimize these off-target DSBs and to show that these systems do not cause oncogenic mutations/translocations using the cell-based and mouse-model systems described earlier. An additional potential problem is that the donor DNA can also integrate in a random fashion. While the donor can be engineered so it is promoter-less and enhancer-less to minimize insertional activation of nearby genes, the random integration of the donor could still potentially inactivate genes (such as tumor suppressors).

We have discussed using rAAV and DSBs as independent ways to stimulate homologous recombination. Two papers have shown that rAAV and DSBs generated using the I-SceI endonuclease can act together to stimulate homologous recombination [55,56]. Studies to determine if rAAV and ZFNs act in a similar manner are ongoing.

## Using Stem Cell Expansion to Make Gene Therapy Safer

An approach to make gene therapy safer is to take advantage of the ability of stem cells to expand. In this scenario, gene transfer would occur in vitro. Individual or small pools of cells would be isolated, expanded, and characterized. Those clones or pools of cells that were determined to have safe and effective integrations would be further expanded, generating enough cells to reinfuse into the patient. For hematopoietic diseases, the major limitation to this approach is the inability to sufficiently expand individual hematopoietic stem cells in vitro. Theoretically, one might generate corrected cells by first using gene targeting in patient-specific human embryonic stem (ES) cells, then expanding the targeted clones in vitro to generate sufficient numbers, followed by differentiation of clones into the tissue-specific stem cells of choice. A proof of principle of this type of gene therapy has been performed in a mouse model of SCID, but several major problems remain [57]. One of the problems for hematopoietic diseases is being able to convert ES cells into hematopoietic stem cells that can give rise to definitive hematopoiesis without having to use transgenes that are associated with leukemias. The general problem of turning human ES cells into tissue-specific stem cells that can then integrate themselves into the host remains great, but the potential utility is enormous, and this remains an exciting area of research that is directly relevant to gene therapy.

## Summary

The seeming conceptual simplicity of gene therapy has belied the difficulty in its actual implementation. Despite waning enthusiasm from the general scientist, gene therapy researchers have persisted in tackling each problem as it has presented. In this review, we have highlighted the next set of problems and some possible solutions for the gene therapy field. We have consciously proposed criteria that set a high bar. We believe, however, that the tortoise had it right: slow and careful is fast, and ultimately we are optimistic that gene therapy will fulfill its promise.

## Abbreviations

CGD: chronic granulomatous disease
DSB: double-strand breaks
ES: embryonic stem
LTR: long terminal repeat
OTC: ornithine transcarbamylase
rAAV: recombinant adeno-associated virus
SCID: severe combined immunodeficiency disease
ZFN: zinc finger nuclease
